# New Insights on Respiratory Syncytial Virus Prevention

**DOI:** 10.3390/vaccines11121797

**Published:** 2023-11-30

**Authors:** Edyta Kopera, Hanna Czajka, Paweł Zapolnik, Artur Mazur

**Affiliations:** College of Medical Sciences, University of Rzeszów, 35-315 Rzeszów, Poland; ekopera@ur.edu.pl (E.K.); pawel.zapolnik@onet.pl (P.Z.); drmazur@poczta.onet.pl (A.M.)

**Keywords:** RSV, prevention, immunoprophylaxis, vaccines, monoclonal antibody

## Abstract

Respiratory syncytial virus (RSV) is a well-known infant pathogen transmitted mainly by droplets. It is a leading cause of upper respiratory tract infections in children, usually with a mild course of illness. RSV has also been a threat to older people, especially those with underlying medical conditions. For a long time, prevention was limited to passive immunoprophylaxis with palivizumab for high-risk infants. There was a strong need to find other treatment or prevention methods against RSV infections. In addition, after the coronavirus disease 2019 (COVID-19) pandemic, some significant changes in RSV epidemiology have been observed. Researchers noticed the shift in RSV seasonality and age distribution and the increased number of cases in older infants and adults. All of these made the need to find other medical options even stronger. Fortunately, two protein-based vaccines against RSV have successfully passed all phases of clinical trials and have been approved for use by adults and older people. One of them is also approved for infants from birth to 6 months of age (after maternal immunisation during pregnancy) and for pregnant women between 24 and 36 weeks of pregnancy. Also, a new passive immunisation option named nirsevimab (a highly potent monoclonal antibody with a long half-life) is now available for the paediatric group. In this review, we will discuss the previous and current RSV prevention methods in the light of structural discoveries of RSV antigens.

## 1. Introduction

Respiratory syncytial virus (RSV) is a common respiratory virus responsible for upper (URT) and lower respiratory tract (LRT) infections, mainly in infants and older adults. Usually, RSV infections, especially of the upper respiratory tract, are mild with symptoms similar to cold or flu. However, the virus can cause severe and acute respiratory illness. Among those particularly vulnerable to a severe course of RSV infection are premature infants and infants with medically complex conditions or weakened immune system, as well as older adults with underlying comorbidities [1,2,3]. For years, RSV treatment options were very limited, especially for the paediatric population. One of the antiviral drugs, named ribavirin (nucleoside), is licensed only for infants with bronchiolitis and is not recommended for routine RSV treatment. For preventing severe RSV infection in children, passive immunoprophylaxis with palivizumab has been available but only for high-risk infants. The coronavirus disease 2019 (COVID-19) pandemic has significantly influenced RSV infections, including the shift in age distribution, altered patterns of RSV seasonality and an increased number of cases, both in older infants and adults. All these phenomena have accelerated the registration of the first RSV vaccines for adults, infants and pregnant women and other treatment options for infants and young children. There are three main target groups for RSV prevention: paediatric, maternal and elderly. The following review will focus on the prevention of RSV infection in the post-COVID-19 era, mainly in children and older adults.

## 2. General Overview of RSV Structure

RSV is a negative-sense, single-stranded ribonucleic acid (RNA) virus with filamentous morphology [4]. The genetic material of the virus is surrounded by an envelope, which is a lipid bilayer derived from the host cell membrane. RSV phylogenetically belongs to the Orthopneumovirus genus of the *Pneumoviridae* family. The 15.2 kbp RSV genome consists of ten RNA segments, which are translated into 11 different proteins. Three of them, named the attachment (G), the fusion (F) and the small hydrophobic (SH) protein, are embedded in the lipid bilayer/membrane. Underneath this membrane, there is a layer of non-glycosylated matrix (M) protein that provides structural support [5]. M protein interacts with M2-1 protein, which creates an intermediate layer and mediates the association between ribonucleocapsid and the matrix protein.

M2-1 is crucial in viral sequential transcription as a transcription antitermination factor [6]. M2-2 protein is generated from another open reading frame (ORF) of the M2 gene and was shown to be involved in the balance between transcription and RNA replication. Interestingly, with the M2-2 knockout mutation, the virus growth is attenuated, but the expression of the gene is increased [7], which is a very attractive phenotype for vaccine development. Other viral proteins include nucleoprotein (N), large polymerase subunit (L) and phosphoprotein, and polymerase cofactor (P). N protein binds tightly to the viral RNA, encapsidating and protecting it within the ribonucleoprotein structure. L protein is involved in RNA synthesis, and P acts as a linker between N and L proteins [5,8]. There are also two non-structural proteins: NS1 and NS2. These proteins are independent pathogenic factors. The first mediates the inhibition and RNA replication, while the second is not essential for RNA replication but improves virus growth in cell culture [9]. Both proteins play a crucial role in suppressing the innate immune response and could be a target of RSV therapy [10]. An in-depth understanding of the virus structure, its genome, and encoded proteins will undoubtedly contribute to the development of effective new-generation therapies.

### The Main Virus Antigens

The RSV G and F glycoproteins are the main virus antigens that are very important for the virus pathogenesis and the host immune response. The attachment G protein (RSV G) plays a crucial role in viral attachment to host cells, facilitating the initial step of viral infection. It is synthesized both as a membrane-bound and secreted form. The first one is responsible for viral attachment through binding to specific receptors (including glycosaminoglycans) on the host cell surface. The second one mediates immune evasion [11,12]. The membrane-bound form is a multi-domain protein. It consists of a short cytoplasmic tail, transmembrane domain, and two mucin-like regions, which are highly glycosylated, and flank two other protein regions: a central conserved domain with a CX3C motif and a heparin-binding domain [13]. The unique feature of the RSV G protein is its glycosylation pattern with ~5 N-linked glycans and ~40 O-linked glycans. It was shown that carbohydrates constitute not less than 50% of the molecular weight of the mature molecule [14,15]. RSV G protein undergoes rapid changes and variations. Antigenic differences in the G protein are the main basis for classifying RSV subtypes [16,17]. Human RSV is classified into A or B subtypes. There are nine various genotypes in RSV-A group (GA1-GA7, SAA1 and NA1) and not less than 32 genotypes in RSV-B (BA1-14, GB1-GB5, SAB1-4, URU1-2, NZB1-2, BA-CCA, BA-CCB, BA-C, CBB and CB1) [18,19]. Viruses of both subtypes commonly co-circulate during the annual epidemic period. However, RSV-A viruses are usually predominant [20,21]. Targeting the attachment protein is a potential approach for anti-RSV drug development and vaccine design. Certainly, the antigenic diversity of the protein is a major challenge to achieving these goals.

The fusion F protein (RSV F) is another main glycoprotein found on the virus surface. It plays a crucial role in the infection process not only by mediating the fusion of the viral envelope with the host cell membrane but also by assisting in virus attachment. Unlike variable RSV G protein, RSV F is highly conserved. It is synthesized as an inactive F0 precursor, post-translationally glycosylated at 5 or 6 N-glycosylation sites and cleaved into two subunits by furin-like host enzymes. The resulting F1 (C-terminal) and F2 (N-terminal) subunits are linked via two disulfide bonds [22]. The proteolytic cleavage of the RSV F protein is required for its activity. The mature protein forms compact trimers on the virus surface [23]. Cleaved and trimerized F glycoprotein adopts a metastable prefusion (RSV pre-F) conformation characterized by a compact structure with the hydrophobic fusion peptide buried within the protein [24]. During the fusion process, the protein undergoes major conformational changes and adopts extremely stable postfusion (RSV post-F) conformation, which represents the final state of the fusion protein [25]. These conformational changes are essential for the successful fusion of the virus particle with the host cell membrane during the infection process. In the RSV post-F conformation, the hydrophobic fusion peptide is exposed at the F1 subunit, allowing it to insert into the host cell membrane.

Moreover, the F1 subunit adopts, at some stage, a hairpin-like structure, bringing the viral and host cell membranes into close proximity. Stabilization of the RSV pre-F conformation revealed significant immunogenic characteristics of the protein. It is the pre-F conformation that elicits highly neutralizing antibodies, which are crucial for blocking virus infection [23,26]. Two of the RSV pre-F epitopes, site ϴ and V become particularly important for the selection of highly potent monoclonal antibodies with therapeutic potential [27]. They are present only on the RSV pre-F protein. The other antigenic sites (I, II, III and IV), which are less immunogenic, are present on both RSV pre-F and post-F forms, except for epitope I, which is present only on post-F [25,28]. The main characteristics of RSV F epitopes are presented in Table 1.

Understanding the structure and conformational changes of the fusion protein was essential for developing antiviral therapies. The RSV pre-F conformation became a crucial antigen for developing an effective RSV vaccine [28,29,30,31,32].

## 3. Epidemiology of RSV Virus after COVID-19 Pandemic

RSV is especially dangerous for premature infants. Also, babies younger than six months of age and children with bronchopulmonary dysplasia, congenital heart disease and immunodeficiency are at risk for RSV-associated severe illness and death [33,34]. In 2019, RSV was responsible for over 30 million lower respiratory infections, 3 million hospitalisations associated with acute respiratory infections and more than 100,000 deaths in young children (0–5 years) globally [33]. More than 95% of RSV-associated acute lower respiratory infection episodes and more than 97% of RSV-attributable deaths across all age bands were in low-income and middle-income countries (LMICs) [33]. The association of early RSV bronchiolitis with recurrent wheezing and asthma has been reported [35]. RSV also imposes a significant risk to older patients (age ≥ 65), especially with underlying comorbidities [2,3,35]. The clinical manifestations of RSV infection in this age group are nonspecific and variable; thus, diagnosis might be problematic [36]. According to late reports from the Centers for Disease Control and Prevention (CDC), due to RSV infections, there are ca. 60,000–160,000 hospitalisations among older adults and 6000–10,000 deaths in this age group annually. RSV also poses a threat to pregnant women [37]. For years, the epidemic curve for RSV was relatively constant. The COVID-19 pandemic has impacted the epidemiology of RSV, although the full extent and implications are still being studied. During the COVID-19 pandemic, epidemiologists reported a marked reduction in influenza, RSV and other respiratory viruses activity in several countries. Implementing non-pharmaceutical interventions such as social distancing and isolation aimed at reducing severe acute respiratory syndrome coronavirus 2 (SARS-CoV-2) transmission also limited the spread of RSV [38,39,40,41]. Currently, in Poland and other countries, an increase in RSV infections is reported, with an increasing number of infections among an older cohort of susceptible children [42,43,44,45]. Also, changes in RSV seasonality patterns have been observed. Typically, the RSV season occurs during the fall, lasts through the winter and ends in the late spring. During the COVID-19 pandemic, the timing and seasonality patterns have been disrupted. Numerous countries, such as Israel, Australia, South Africa, New Zealand, Italy, France, the United States and Japan, have experienced unexpected seasonality shifts [38,43,46,47]. The temporary decline in RSV circulation during the COVID-19 pandemic may have led to an increase in the number of susceptible individuals in the population, including older children and adults who may have had limited exposure to RSV in recent months. This accumulation of susceptible individuals potentially results in more significant outbreaks when RSV activity resumes.

## 4. Immunoprophylaxis

There are two general methods of immunoprophylaxis: passive and active immunisation, however, in the case of RSV infection, for years, only passive immunisation was available and for high-risk infants, exclusively. We are currently witnessing a breakthrough with the registration of the first two RSV vaccines for older adults and a new monoclonal neutralising antibody with a much longer half-life for infants and young children. In the near future, we can certainly expect new RSV interventions in the field of passive and active immunisation since there are several products in clinical trials. An important aspect of immunoprophylaxis is the vaccination of pregnant women [48]. The strategy to protect infants through transplacental transfer of vaccine-induced maternal antibodies is under development for RSV prevention. In the following sections, the two methods of RSV immunoprophylaxis will be discussed, both current status and prospects for the future.

### 4.1. Passive Immunisation Using Monoclonal Antibodies

The first passive immunoprophylaxis used in RSV infections was the intravenous administration of immune globulin RSV-IGIV (RespiGam, MedImmune, Gaithersburg, MD, USA). The product consisted of polyclonal antibodies derived from human plasma, rich in the neutralizing anti-RSV activity. RespiGam was dedicated to premature infants and young children with chronic lung disease; however, the treatment exhibited a moderate efficacy and generated high costs. Discoveries in the field of the molecular structure of the RSV F protein, together with the advances in the isolation of monoclonal antibodies, resulted in the development of palivizumab. Palivizumab (Synagis, AstraZeneca, Cambridge, UK) is a humanized murine anti-IgG1κ monoclonal antibody, which is produced via DNA technology in mouse myeloma cells. It targets the highly conserved epitope *II* on the RSV F protein (pre-F and post-F) and possesses fusion-inhibiting effects on the virus (RSV A and B subtypes). It is licenced as an immunoprophylaxis for preterm infants (born at ≤35 weeks of gestation) and infants younger than six months of age when the RSV infection season begins, also for children younger than two years with bronchopulmonary dysplasia or haemodynamically significant heart disease [49]. The need for monthly administration during the period of expected risk of RSV infection and relatively high cost are the heavy burden for health services and the main barriers to the extension of indications. Caserta et al. thoroughly discussed in their recently published report all these aspects and other more concerning palivizumab immunoprophylaxis [50].

There is a need for the next generation antibodies with a longer half-life and higher efficacy. Two monoclonal antibodies hoped to fulfil this need—suptavumab and motavizumab. The first one (MEDI-8897, targeting epitope V on the pre-F) successfully passed Phase I and II but was discontinued after receiving results from the Phase III study in infants. Suptavumab prevented RSV A infections while increasing the rate of RSV B. It did not reduce overall RSV hospitalisations and outpatient lower respiratory tract infections [51]. The second one, named motavizumab (MEDI-524), was derived from palivizumab and targeted the same antigenic site—epitope II. Motavizumab had 20 times higher in vitro neutralisation activity compared to palivizumab and a longer half-life [52]. Regardless of these attractive features, its clinical trial was discontinued due to increased incidence of hypersensitivity reactions. In the meantime, a monoclonal antibody D25, which selectively binds to the antigen *ϴ* site of the RSV pre-F, was isolated. Through binding to this specific epitope, the antibody is able to block protein conversion to the postfusion form at the stage of the prehairpin intermediate. The antibody was further optimised by introducing mutations (YTE) in the Fc region, which allowed to obtain the variant with a longer half-life and enhanced potency. This antibody variant named nirsevimab (Beyfortus, AstraZeneca, Cambridge, UK, and Sanofi, Paris, France) became the second monoclonal antibody (fully human) licensed for infant immunoprophylaxis. It is recommended for the prevention of RSV lower respiratory tract disease in infants and children up to 24 months of age with a single injection for protection throughout the RSV season. In the study among healthy late preterm and full-term infants, nirsevimab showed an efficacy of 75% against RSV lower respiratory tract infections and a reduction in RSV-related hospitalisations compared to placebo [53]. In another study among preterm infants and infants at higher risk for severe RSV infection (congenital heart and lung diseases), the efficacy and safety of nirsevimab were compared to palivizumab. The study showed a similar efficacy and safety profile to that of palivizumab [54,55]. The latest results from the Phase IIIb HARMONIE (international clinical trial) study demonstrated an 83.2% reduction in hospitalisations due to RSV-related LRT infection in infants under 12 months who received nirsevimab, compared to placebo. Nirsevimab also reduced the incidence of hospitalisations due to severe RSV-related LRT disease by 75.71% in infants under 12 months compared to placebo [56].

Clesrovimab (MK-1654) is another monoclonal antibody (fully human) with a long half-life and proven safety [57]. It targeted highly conserved site *IV* of the RSV F protein. Clesrovimab is currently in Phase IIb/III to evaluate the efficacy and safety in healthy pre-term and full-term infants versus placebo (NCT04767373) and in Phase III to evaluate the safety, efficacy and pharmacokinetics in infants and children at increased risk for severe RSV disease compared to palivizumab (NCT04938830). To reduce the costs of passive immunisation, other products are under development. The first one is a Ø site monoclonal antibody named RSM01. It is currently in a Phase I trial (NCT05118386) to evaluate the safety, tolerability and pharmacokinetics of single ascending doses in healthy adults. The second one is TNM001 (Trinomab Biotech, Zhuhai, China). It is a fully human monoclonal antibody which acts by targeting the RSV F protein. Currently, in Phase Ib/IIa study (NCT05630573), the safety, tolerability and pharmacokinetics profile of TNM001 injections in Chinese healthy and term infants is being assessed. A summary of monoclonal antibodies used for RSV prophylaxis is presented in Table 2.

### 4.2. Active Immunisation

Since the first isolation of RSV from chimpanzees with coryza, various studies have been conducted to develop an effective vaccine. The process was long and full of humble discoveries of the virus’s nature. Initially, well-tolerated formalin-inactivated RSV vaccine tested in children eventually gave aberrant results, with the death of two patients. It was found that inactivated vaccines evoked too few virus-blocking antibodies but a major inflammatory response, including pulmonary neutrophil infiltration [58]. These caused difficulties in RSV vaccine development due to obvious safety concerns. The development process was challenging due to the unique characteristics of the virus. The breakthrough in the field came after the stabilisation of the RSV pre-F conformation. Recently, two vaccines, both based on the RSV F in its pre-F form, were approved by the Food and Drug Administration (FDA). One of them is AREXEVY (GlaxoSmithKline, Brentford, UK), which has also been approved in the European Union by the European Medicines Agency (EMA). It is a monovalent adjuvanted vaccine, which contains 120 µg of recombinant RSV pre-F glycoprotein (RSVPreF3 antigen) and AS01E as an adjuvant for intramuscular injection. The RSVPreF3 antigen is produced in Chinese hamster ovary (CHO) cells using recombinant DNA technology and then thoroughly purified in a multi-step process. The AS01E adjuvant was selected in the Phase I/II trial when the safety and immunogenicity of the vaccine were studied in adults (*N* = 48, aged 18–40 years) and older adults (*N* = 1005, aged 60–80 years). The vaccine proved to be safe, and AS01E adjuvant was superior to AS01B [59]. In Phase III, participants received one dose of RSVPreF vaccine (*N* = 12,467) or placebo (*N* = 12,499). Vaccine efficacy against RSV-related lower respiratory tract disease, severe RSV-related LRT disease and RSV-related acute respiratory infection was shown to be 82.6%, 94.1% and 71.1%, respectively. Vaccine efficacy against infections of RSV subtypes A and B was similar. The safety profiles were proved regardless of underlying medical conditions [60].

The second approved vaccine is ABRYSVO (Pfizer, New York, NY, USA) bivalent vaccine. This vaccine represents a different strategy—a final formulation containing RSV pre-F antigens from both RSV subtypes A and B without any adjuvant. The ABRYSVO vaccine contains in total 120 µg of protein (60 µg RSVpreF A and 60 µg RSVpreF B) for single intramuscular injection. Both proteins are expressed in CHO cells and then purified through a series of column chromatography and filtration steps. In Phase IIa (healthy adults, 18 to 50 years), the vaccine showed 86.7% efficacy for symptomatic RSV infection confirmed by any detectable viral RNA on at least two consecutive days (NCT05035212, RENOIR) [61]. In Phase 3, the efficacy, immunogenicity, and safety of a single dose of the vaccine were studied in adults 60 years of age and older. Participants had received RSVpreF vaccine (*N* = 17,215) or placebo (*N* = 17,069). The authors showed that vaccine efficacy for preventing RSV-associated LRT illness and RSV-associated acute respiratory illness was 85.7% and 62.1%, respectively [62]. Both already registered vaccines have been further studied for other clinical purposes. The RSVpreF vaccine is checked for immunogenicity, safety and reactogenicity when co-administered with the influenza vaccine (Fluzone High-Dose Quadrivalent, Sanofi, Paris, France) in adults aged 65 and older compared to separate administration of both vaccines (NCT05559476, NCT05568797). Also, the bivalent RSVpreF vaccine is studied to evaluate the safety, tolerability and immunogenicity when co-administered with seasonal inactivated influenza vaccine in adults aged 65 and older (NCT05301322). Lately (July 2023), the bivalent RSVpreF vaccine (ABRYSVO) was approved by EMA for infants from birth to 6 months of age (after maternal immunisation during pregnancy) and for pregnant women between 24 and 36 weeks of pregnancy. It is the first vaccine against RSV approved to protect pregnant women by active immunization and their infants in consequence by passive immunisation. It was approved after receiving the results from the Phase III clinical study. This was run to study the efficacy and safety of maternal immunisation against medically attended LRT illness (MA-LRTI). The efficacy, safety and immunogenicity were assessed in infants born to healthy women who received the vaccine while being pregnant. Simultaneously, the safety and immunogenicity in the pregnant women were evaluated (NCT04424316). In this study, 3682 pregnant women received the vaccine, and 3676 received a placebo, then 3570 (vaccinated mothers) and 3558 infants (placebo mothers) were tested. The results showed that vaccine efficacy for preventing medically attended severe LRT illness within 90 and 180 days after birth was 81.8% and 69.4%, respectively [63]. Under clinical development is another maternal vaccine, which is based on RSVpreF antigen. In the Phase IIb study, the safety, tolerability, and immunogenicity of an RSVpreF subunit vaccine (RSV vaccine) are evaluated in pregnant women who receive either one of two doses of the vaccine, formulated with/without aluminium hydroxide or placebo, as well as the safety and characteristics of transplacentally transferred antibodies in their infants (NCT04032093). In this clinical study, 406 women and 403 infants were included, and 327 women (80.5%) received the RSVpreF vaccine. The RSVpreF vaccine proved to elicit neutralising antibody responses with efficient transplacental transfer [64]. 

The COVID-19 pandemic undoubtedly brought a breakthrough in the field of vaccines since for the first time in the history, vaccines based on nucleic acids such as mRNA vaccines were used. The possibility of using this type of vaccines was the result of many years of brilliant scientific research, which has been honoured this year with the Nobel Prize. The most advance mRNA vaccine against RSV is the mRNA-1345 vaccine developed by Moderna. The vaccine is adjuvanted with LNP and aim to produce RSV F protein. It is currently under Phase III of clinical trials [NCT05330975]. The study is divided into Part A, B and C. The purpose of Part A study is to evaluate the safety, tolerability and immunogenicity of mRNA-1345 co-administered with a seasonal influenza vaccine (Alfuria^®^, quadrivalent). The second purpose is to evaluate both the impact of co-administered influenza vaccine on the immune response to RSV-A and the impact of RSV vaccine on the immune response to influenza. The purpose of Part B study is to evaluate the safety, tolerability and immunogenicity of mRNA-1345 co-administered with mRNA-1273.214. The other purpose of the study is to evaluate both the impact of co-administered mRNA-1273.214 vaccine on the immune response to RSV-A and to evaluate the effect of the RSV vaccine on the immune response to SARS-CoV-2. Finally, the purpose of Part C study is to evaluate the safety and tolerability of booster dose of mRNA 1345 administered one year after a primary dose. It is a continuation of the study after obtaining positive results from Phase I (NCT04528719) when the tolerability and reactogenicity of a single injection of up to five dose levels of mRNA-1345 in younger adults, women of child-bearing potential and older adults were assessed, and from Phase II (NCT05127434) when the efficacy of a single dose of mRNA-1345 vaccine in the prevention of a first episode of RSV-associated lower respiratory tract disease as compared with a placebo was assessed. The mRNA-1345 vaccine is also studied in children who are (RSV)-seropositive, as a Phase I of clinical trial (NCT04528719). In the paediatric group, another mRNA vaccine is studied. This is the mRNA-1365 vaccine developed by Sanofi. Phase I trials have recently begun aim to assess the safety and immunogenicity of mRNA-1365, an mRNA vaccine targeting RSV and human metapneumovirus (hMPV), and mRNA-1345 in infants aged 5 months to <24 months (NCT05743881). 

Currently, several other experimental vaccines are under clinical trials, including recombinant subunit vaccines, live attenuated vaccines and viral vector-based vaccines (see Table 3). The latter may soon be approved for use. 

## 5. Conclusions

RSV prevention has been a challenge for many years. Recently licenced new passive (Nirsevimab) and active (AREXEVY and ABRYSVO) prevention options and the results from maternal immunisation demonstrate that there is hope for broader medical choices, which will be available for paediatric groups and adults. 

## Figures and Tables

**Table 1:** RSV F epitopes and their main characteristic.

Site Name	RSV F Conformation	Characteristic	Product
θ	pre-F	α-helix from F1 (aa 196–210) and a strand from F2 (aa 62–69), D25-specific antibody, AM22-specific antibody	Nirsevimab, RSM01 (under clinical development)
I	post-F	located at F2 subunit, Pro389, 131-2a	-
II	pre-F and post-F	helix-turn-helix motif from F1 (aa 253–278), conserved epitope, Mota-specific antibody	Palivizumab, Motavizumab (discontinued)
III	pre-F and post-F	two anti-parallel β-strands, MPE8-specific antibody	-
IV	pre-F and post-F	linear epitope (aa 422–436), 101F-specific antibody, does not blocking virus attachment.	Clesrovimab (under clinical development)
V	pre-F	α2-α3 and β3-β4, located between site Ø and III on the RSV pre-F, AM14-specific antibody	Suptavumab (discontinued)

Abbreviations: RSV—respiratory syncytial virus.

**Table 2:** RSV immunoprophylaxis by target populations. Monoclonal antibodies are categorised into sequences of clinical phases (I, II, III) for two different population groups: paediatric and adults.

Population	Phase I	Phase II	Phase III	Market Approved
Children	-	-	Clesrovimab (MK-1654) fully human mAb (IgG1) binding RSV F protein site *IV*, half-life: 73–88 days, single injection	Palivizumab Humanized mAb (IgG1) binding RSV F protein site II, half-life: 20 days, monthly intramuscular injection
	TNM001 human anti-RSV mAb injection	-	-	Nirsevimab fully human mAb (IgG1) binding RSV F protein site *Ø*, YTE mutation of the Fc, half-life: 65–70 days, single intramuscular injection
Adults	RSM01 Humanized mAb (IgG1) binding RSV F protein site *Ø*, half-life: 65–70 days, single injection	-	-	-

Abbreviations: IgG1—immunoglobulin G subclass 1; mAb—monoclonal antibody; RSV—respiratory syncytial virus.

**Table 3:** RSV vaccines by target populations. Vaccine candidates are categorised into sequences of clinical phases (I, II, III) for different population groups: paediatric, maternal and adults/elderly. Various type of vaccines are marked with colours: blue refers to recombinant subunit vaccine; green: live attenuated; orange: mRNA; red: recombinant viral vector; grey: virus-like particle (VLP).

Population	Phase I	Phase II	Phase III	Market Approved
Children	PIV5-vectored RSV Vaccine (BLB-201) (NCT05655182) live attenuated chimeric, antigen: RSV fusion F, intranasal route MV-012-968 (NCT04909021, NCT04444284), live, attenuated target organism, antigen: all viral proteins, intranasal route LID/ΔM2-2/1030s (NCT04520659), live, attenuated target organism, antigen: all viral proteins except M2-2, intranasal route RSV NS2/Δ1313/I1314L, RSV 6120/ΔNS2/1030s, RSV 276 (NCT03916185), live attenuated, antigen: all viral proteins except NS2, intranasal route RSV6120/∆NS1, RSV6120/F1/G2/∆NS1 (NCT03596801), live attenuated, antigen: all viral proteins except NS2, intranasal route mRNA-1345 (NCT04528719), antigen: RSVF mRNA-1365 mRNA-1345 (NCT05743881) human metapneumovirus (hMPV antigen: RSVF	VAD00001 (NCT04491877), live, attenuated, antigen: all viral proteins, intranasal route	-	ABRYSVO^®^ recombinant subunit, antigen: RSV preF A and RSV preF B
Pregnant	-	RSV (NCT04032093), recombinant subunit, antigen: RSV F, with aluminium salt RSV vaccine given together with Tdap (NCT04071158), recombinant subunit, antigen: RSV F, with aluminium salt	RSVpreF (NCT04424316), recombinant subunit, antigen: RSV preF	ABRYSVO^®^ recombinant subunit, antigen: RSV preF A and RSV preF B
Adults and Elderly	CPI-RSV-F Vaccine (NCT05281263) live attenuated chimeric, antigen: RSV fusion F, intranasal route CodaVaxRSV (NCT04295070), live attenuated, antigen: codon deoptimized RSV, intranasal route IVX-A12 (RSV/hMPV) (NCT05664334), bivalent combination respiratory syncytial virus (RSV)/human metapneumovirus (hMPV) virus-like particle (VLP) vaccine, with MF59 adjuvant V-306 (NCT04519073), synthetic virus-like particle (SVLP) vaccine, with Pam2Cys adjuvant mRNA-1345, mRNA-1045, mRNA-1230 (NCT05585632), multi-component, antigen: RSVF/Flu-HA/SARS-CoV-2S mRNA LNP (NCT05639894), vaccine candidate formulated with two different lipid nanoparticles (LNPs) CL-0059 and CL-0137 rBCG-N-hRSV (NCT03213405) recombinant *Mycobacterium bovis* BCG vaccine that expresses the human RSV nucleoprotein (N)	BARS13 (NCT04681833) recombinant subunit, antigen: rRSV-G-protein, with CsA adjuvant VN-0200 (NCT05547087), recombinant subunit, antigen: VAGA-9001a, with MABH-9002b adjuvant RSVPreF (NCT04785612), recombinant subunit, antigen: RSV F RSVPreF together with SIIV (NCT03529773), recombinant subunit, antigen: RSV F, with aluminium salt MV-012-968 (NCT04690335), live, attenuated target organism, antigen: all viral proteins, intranasal route mRNA-1345 (NCT05127434), antigen: RSVF	MVA-BN-RSV (NCT05238025) recombinant viral vector, antigen: two surface proteins and internal conserved protein Ad26.RSV.PreF (NCT05070546, NCT05071313, NCT05083585, NCT05101486, NCT0524243, NCT05327816), live attenuated, antigen: RSV fusion F mRNA-1345 (NCT05330975), antigen: RSVF, co-administered with a seasonal influenza vaccine (Afluria^®^ Quadrivalent)	AREXEVY^®^ recombinant subunit, antigen: RSVPreF3, with AS01E adjuvant ABRYSVO^®^ recombinant subunit, antigen: RSV preF A and RSV preF B

Abbreviations: BCG—Bacillus Calmette–Guérin; hMPV—human metapneumovirus; LNP—lipid nanoparticle; mRNA—messenger ribonucleic acid; RSV—respiratory syncytial virus; SVLP—synthetic virus-like particle; Tdap—diphtheria, tetanus, pertussis vaccine with lower concentration of diphtheria and pertussis toxoids; VLP—virus-like particle.

## Data Availability

Not applicable.

## Abbreviations

BCG	Bacillus Calmette–Guérin
CDC	Centers for Disease Control and Prevention
CHO	Chinese hamster ovary
COVID-19	Coronavirus disease 2019
EMA	European Medicines Agency
FDA	Food and Drug Administration
hMPV	human metapneumovirus
IgG1	Immunoglobulin G subclass 1
kbp	Kilo-base pairs
LNP	lipid nanoparticle
LRT	Lower respiratory tract
mAb	Monoclonal antibody
MA-LRTI	Medically attended LRT illness
mRNA	Messenger ribonucleic acid
ORF	Open reading frame
RSV	Respiratory syncytial virus
RNA	Ribonucleic acid
SARS-CoV-2	Severe acute respiratory syndrome coronavirus 2
SVLP	Synthetic virus-like particle
Tdap	Diphtheria, tetanus, pertussis vaccine with lower concentration of diphtheria and pertussis toxoids
URT	Upper respiratory tract
VLP	Virus-like particle.
